# Characterization of *Salmonella* Resistome and Plasmidome in Pork Production System in Jiangsu, China

**DOI:** 10.3389/fvets.2020.00617

**Published:** 2020-09-11

**Authors:** Qingxin Liu, Wenjing Chen, Mohammed Elbediwi, Hang Pan, Liqun Wang, Chuang Zhou, Bin Zhao, Xinguo Xu, Dingguo Li, Xin Yan, Xiao Han, Hanyuan Li, Yan Li, Min Yue

**Affiliations:** ^1^School of Animal Husbandry and Veterinary Medicine, Jiangsu Vocational College of Agriculture and Forestry, Jurong, China; ^2^Department of Veterinary Medicine, Institute of Preventive Veterinary Sciences, Zhejiang University College of Animal Sciences, Hangzhou, China; ^3^Zhejiang Provincial Key Laboratory of Preventive Veterinary Medicine, Hangzhou, China

**Keywords:** *Salmonella*, antimicrobial resistance, resistome, plasmidome, pig slaughterhouse, cross-contamination

## Abstract

The prevalence of antimicrobial resistance in zoonotic *Salmonella* is a significant ongoing concern over the world. Several reports have investigated the prevalence of *Salmonella* infections in the farm animals in China; however, there is only limited knowledge about the *Salmonella* cross-contamination in the slaughterhouses. Moreover, the application of genomic approaches for understanding the cross-contamination in the food-animal slaughterhouses is still in its infancy in China. In the present study, we have isolated 105 *Salmonella* strains from pig carcasses and environment samples collected from four independent slaughterhouses in Jiangsu, China. All the *Salmonella* isolates were subjected to whole genome sequencing, bioinformatics analysis for serovar predictions, multi-locus sequence types, antimicrobial resistance genes, and plasmid types by using the in-house Galaxy platform. The antimicrobial resistance of *Salmonella* isolates was determined using a minimal inhibitory concentration assay with 14 antimicrobials. We found that the predominant serovar and serogroup was *S*. Derby and O:4(B), with a prevalence of 41.9 and 55%, respectively. All the isolates were multidrug-resistant and the highest resistance was observed against antimicrobials tetracycline (95.4%) and trimethoprim and sulfamethoxazole (90.9%). Additionally, the colistin-resistant determinant *mcr*-1 gene was detected in five (4.8%) strains. Our study demonstrated the prevalence of antimicrobial resistance in *Salmonella* strains isolated from pig slaughterhouses in China and suggested that the genomic platform can serve as routine surveillance along with the food-chain investigation.

## Introduction

In the past few years, *Salmonella* infection has increased rapidly in humans and animals, and it is one of the leading cause of foodborne illness-related hospitalization in both developed and developing countries (1–10). In Canada, *Salmonella* was the second most common cause of bacterial foodborne illness outbreaks. *Salmonella* was recovered from 94% of carcass swabs after bleeding and 10% of carcass swabs after de-hairing from two pork processing plants in Alberta, Canada (11). In Africa, *Salmonella* was detected in 13.1% (95% CI 9.3–18.3) of 5,467 pig samples (12). In the past few decades, the prevalence of *Salmonella* infection has increased rapidly, especially in China (13). With the rapid economic growth of China, meat consumption has increased from 43.8 to 54.3 kilograms per person in 2011–2019. Among this, pork remains Chinese favorite meat, which makes up around 60% of total meat consumption, and the consumption of pork is expected to increase by at least 25% in the next decade (14).

Although many studies have investigated the prevalence of *Salmonella* infection in farms (15–17), genomic characterization of *Salmonella* infection in slaughterhouses are still lagging in China. Since pigs from different farms are mixed in the slaughterhouses, *Salmonella* from the infected farms could spread among the herds in the slaughterhouses. Also, the *Salmonella* shedding may enhance due to the stress and various other factors, threatening the safety of food-chain. Thus, an improved understanding is essential to evaluate the risk of *Salmonella* contamination in the slaughterhouses. Furthermore, significant advancements have been achieved in understanding and prediction of antimicrobial resistance of the *Salmonella* (11, 14, 18, 19), and the knowledge of the antimicrobial resistance genes and plasmids are improving. Genomic sequencing could provide unprecedented information about the genetic makeup of individual bacterial pathogens. Combined with the sophisticated bioinformatics pipelines, we can routinely predict the bacterial agent, serovar, antimicrobial resistance determinant, and companion plasmids.

To proof of concept, we have applied whole genome sequencing to analyze 105 *Salmonella* strains recovered from 1,005 samplings from pig slaughterhouses in Jiangsu Province, China. We found that the antimicrobial resistance was relatively high in the slaughterhouses, and the dominant serovar was Derby. Our study provides a genomic view of understanding the prevalence of *Salmonella* in the pig slaughterhouses in China.

## Materials and Methods

### Bacteria Collection and Source Information

A total of 105 *Salmonella* isolates, were recovered from pig slaughterhouses in four cities in Jiangsu province, China, between March and July in 2017. Among the 105 strains, 52 isolates were recovered from pig carcasses, and 53 isolates were recovered from the slaughterhouse environment. The distribution of the strains in different cities was as follows: 43 isolates from Zhenjiang, 27 isolates from Suzhou, 24 isolates from Wuxi, and 11 isolates from Changzhou.

### Culture and Identification

The primary culture and isolation of the organism were performed following the methods recommended by the World Organization for Animal Health Terrestrial Manual (20). Briefly, the samples were pre-enriched in buffered peptone water (BPW) for 18–24 h at 37°C. Then, the samples were cultured using modified semisolid Rappaport Vassiliadis medium for 24 h at 41.5°C. After that, strains were finally isolated by picking up a typical black center single clone on XLT-4 (Xylose Lysine Tergitol-4) agar plates. Then, the strains were identified by PCR using the following primers: *Psa*l-F: 5′-ACAGTGCTCGTTTACGACCTGAAT-3′, *Psa*l-R: 5′-AGACGACTGGTACTGATCGATAAT-3′ (21).

### Antimicrobial Susceptibility Test

The *Salmonella* strains were subjected to antimicrobial minimum inhibitory concentration (MIC) assay using the broth microdilution method, which was described previously (14). Fourteen antimicrobials belonging to ten classes were used for the MIC assay. The cut-off values recommended by CLSI 2017 guidelines (22) were used for the categorization of results, and the intermediate strain, if available, was merged with the resistant strains for the ease of analysis. The MIC range (mg/L) of antimicrobials used in this assay were as follows: ampicillin (AMP): 0.5–64; amoxicillin and clavulanate potassium (AMC): 0.5–64; gentamicin (GEN): 0.25–32, kanamycin (KAN): 0.5–64, streptomycin (STR): 0.5–64; tetracycline (TET): 0.5–64; ciprofloxacin (CIP): 0.015–8; nalidixic (NAL): 0.5–64; chloramphenicol (CHL): 0.5–64; ceftiofur (CF): 0.015–8; cefoxitin (CX): 0.5–64; trimethoprim and sulfamethoxazole (TST): 0.06–32; azithromycin (AZI): 0.5–64; and imipenem (IMP): 0.015–8. We Strains resistant to more than three classes of antimicrobials were defined as multi-drug resistant (MDR). The Two quality control strains, including E. coli ATCC 25922 and Pseudomonas aeruginosa ATCC 27853, were used to validate the antimicrobial susceptibility testing.

### *In silico* Bioinformatics Analysis

The genomic DNA of 105 samples was done as according to pervious study (23–25) using a commercial genomic DNA isolation kit (Tiangen, China). Then, the whole genome sequencing was performed by next-generation sequencing using BGI BGISEQ-500 Platform. The raw sequence reads were under quality check and assembled by using SPAdes v3.12.0 (26). *Salmonella in silico* serotyping was conducted by SISTR web tool. Further, the sequence type, antimicrobial resistance genes (ARG) and plasmid types were detected using assemblies of the samples on the in-house Galaxy platform (27–29), combined with mlst v2.16.1 (https://githubcom/tseemann/mlst2016) and abricate v0.8 (30), including CGE ResFinder database (updated in 2019-04-23) with a similarity cutoff of 90% for ARG and PlasmidFinder database (updated in 2019-04-23) with a similarity cutoff of 95% (31).

## Results

### Prevalence and Serovar Distribution of *Salmonella* Isolates

**Figure 1:**
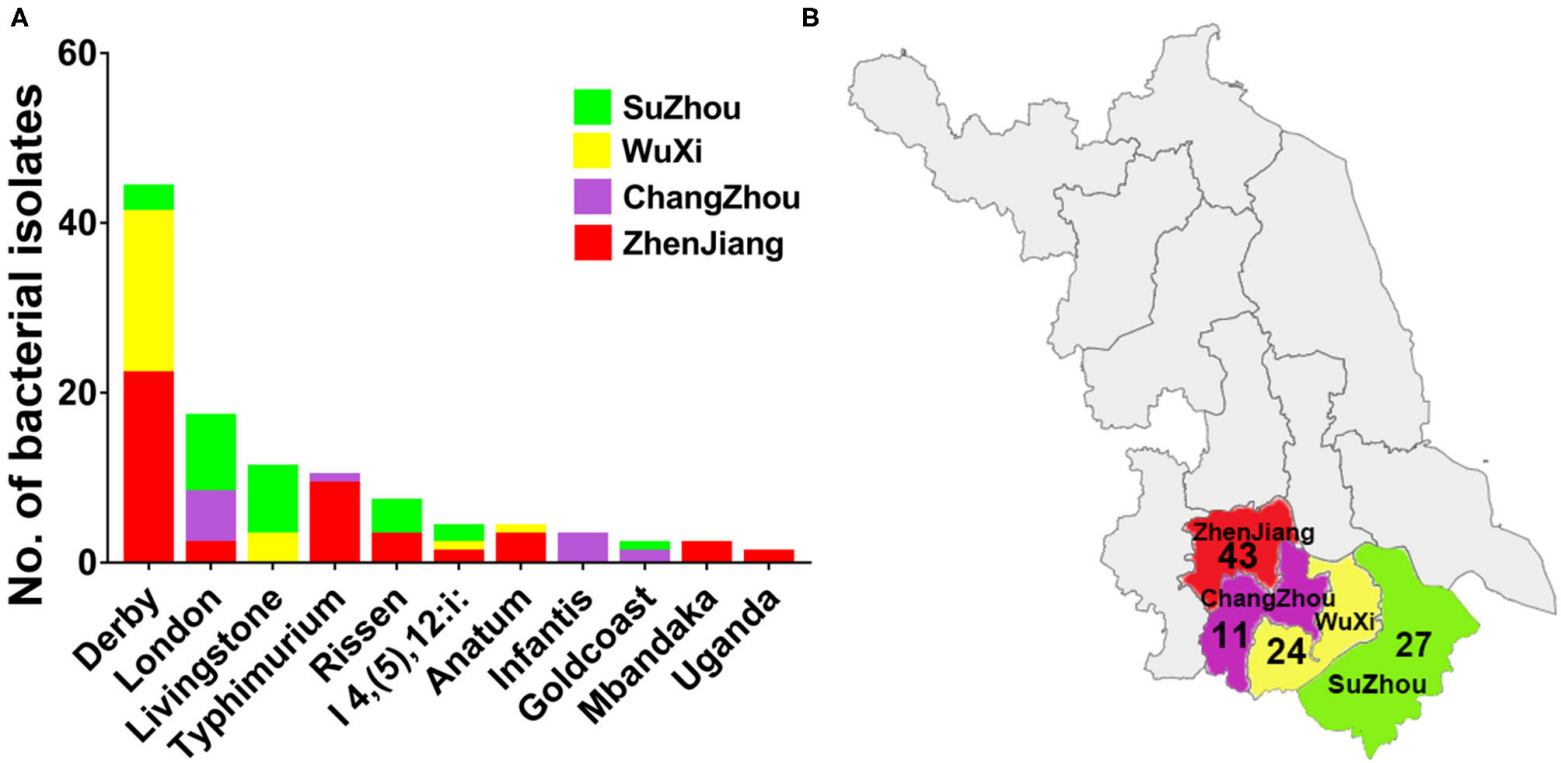
The distribution of different serovars among four cities in Jiangsu province, China. **(A)** The distribution of eleven serovars of *Salmonella* isolates. The dominant serovars are Derby, London and Livingstone. **(B)** The map of Jiangsu province with four cities (Zhenjiang, Changzhou, Wuxi, and Suzhou) which were examined in current investigations. The number below the city name indicates the numbers of isolates collected from individual city.

In this study, 105 non-duplicated isolates were obtained from pig slaughterhouses located in four cities in Jiangsu, China, between March to July in 2017. Among these 105 strains, 52 isolates were recovered from pig carcasses, and 53 isolates were recovered from the environment. As shown in Figure 1, by using microbiological identification, 105 strains were confirmed to be *Salmonella*. The distribution of these strains was as follows: 43 strains from Zhenjiang, 27 strains from Suzhou, 24 strains from Wuxi, and 11 strains from Changzhou. *In silico* serotyping of the isolated strains by using SISTR web tool was revealed that eleven different types of serovars was identified. The prevalence of these serovars were as follows: Derby (41.9%), London (18%), Livingstone (10.4%), Typhimurium (9.5%), Rissen (6.6%), I 4, [5]12:i:- (3.8%), Anatum (3.8%), Infantis (2.8%), Goldcoast (2.8%), and Uganda (0.9%). The dominant serovars were Derby, London and Livingstone.

### Multidrug Resistance Pattern in Serovars and Serogroups

**Figure 2:**
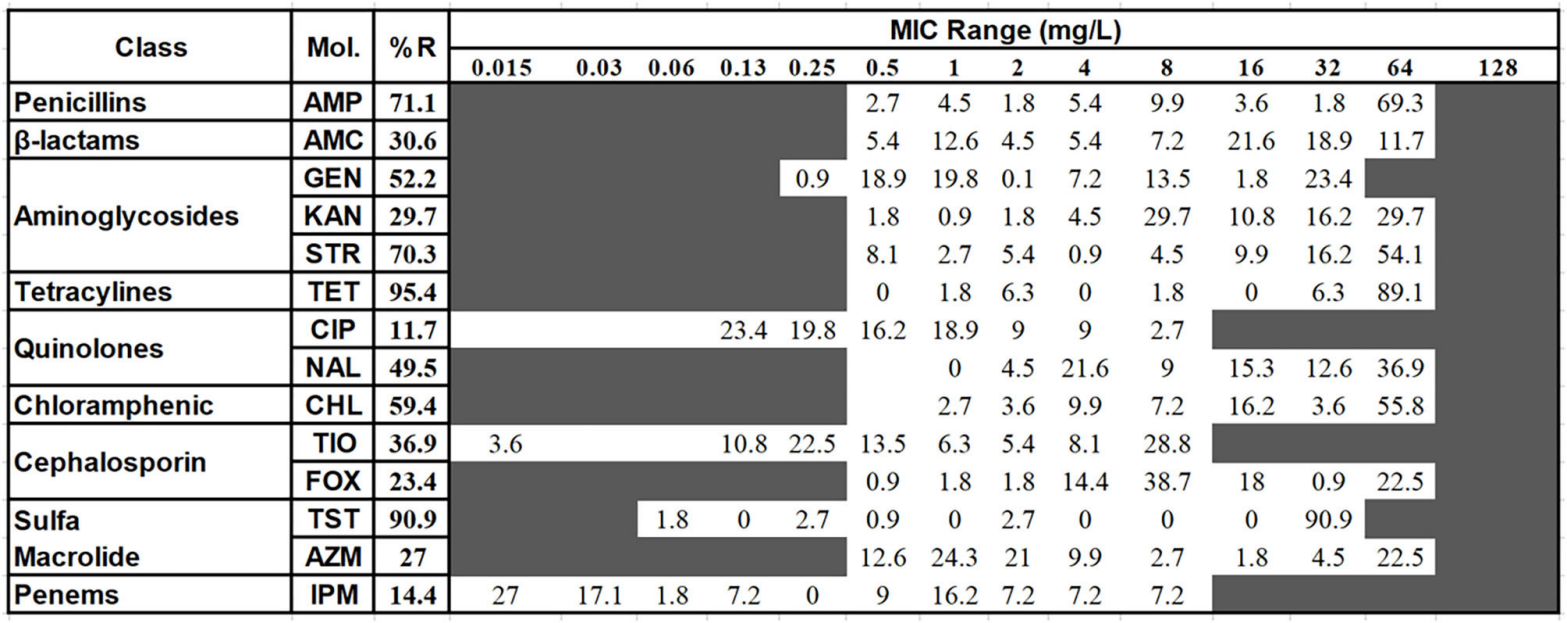
The distribution of the various minimum inhibitory concentration (MIC) values of the examined *Salmonella* isolates against 14 kinds of antimicrobials. The names of the antimicrobials (second column) are abbreviated as AMP, ampicillin; AMC, amoxicillin and clavulanate potassium; GEN, gentamicin; KAN, kanamycin; STR, treptomycin; TET, tetracycline; CIP, Ciproflocacin; NAL, nalidixic; CHL, chloramphenicol; CF, ceftiofur; CX, cefoxitin; TST, trimethoprim and sulfamethoxazole; AZI, azithromycin; IMP, impenem. The third column shows the average resistance (in percentage). The fourth column is the range of the MIC tested. The values in each cell show the percentage of strains in that particular MIC dilution level. The vertical bar indicates the cutoff level of the minimum inhibitory concentration for each antimicrobial at the highest value of that particular cell's dilution for susceptibility (equal to or less than) and resistance (greater than). For better clarification and the ease of analysis, the antimicrobial intermediate group was combined with the resistant group.

The broth microdilution minimum inhibitory concentration (MIC) assay was performed to evaluate the drug resistance of *Salmonella* isolates. The result of the MIC assay was shown in Figure 2. The most severe resistance was observed against the antimicrobials TET (95.4%) and TST (90.9%). Low resistance rates were recorded against CIP (11.7%) and IMP (14.4%).

### Antimicrobial Resistance Among Serovars and Serogroups

**Figure 3:**
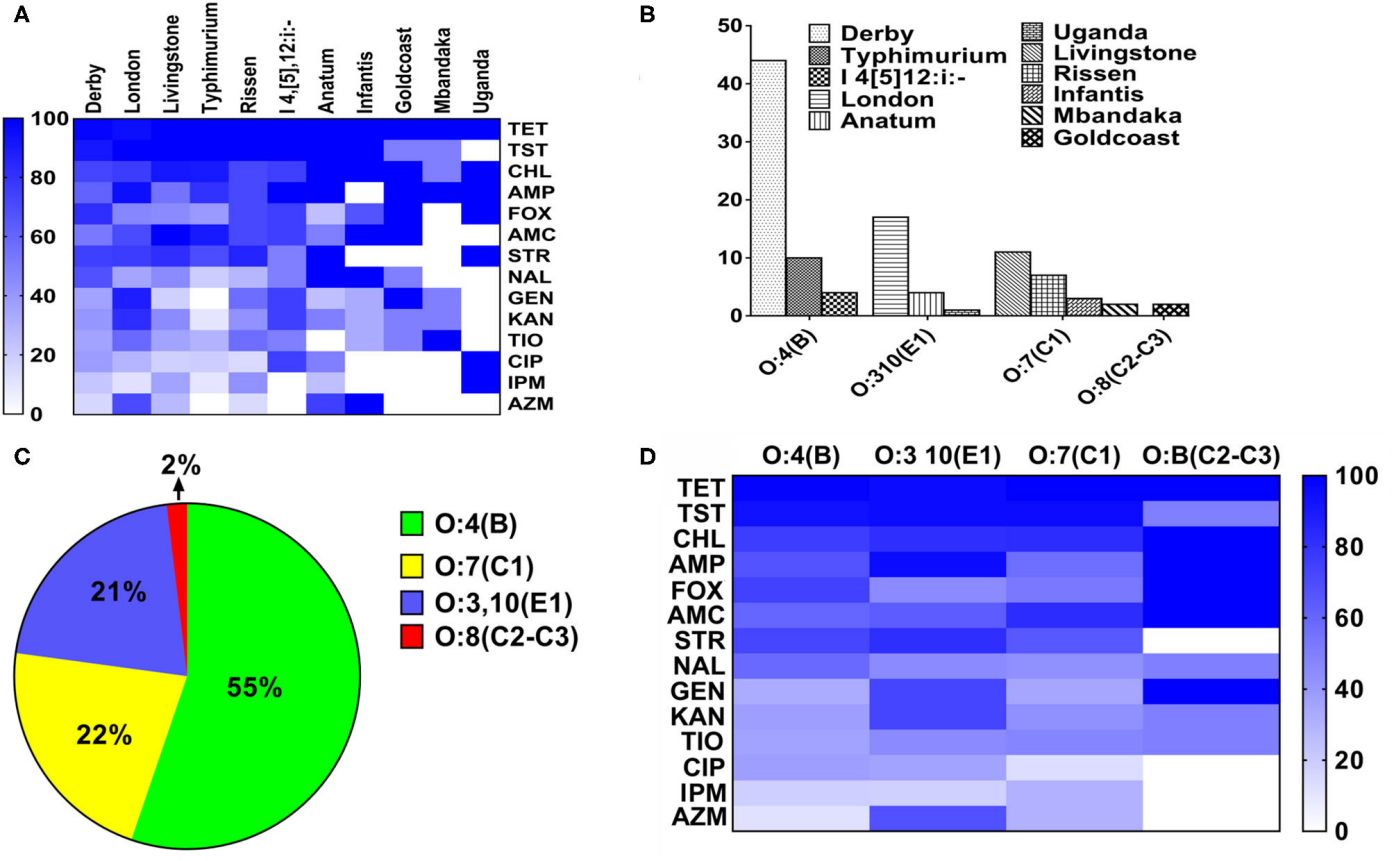
The heatmap of antimicrobial resistance in different serovars and serogroups in *Salmonella*. **(A)** The heatmap of antimicrobial resistance in different serovars in *Salmonella*. The resistance percentage were higher than 95% with TET, TST, and CHL in all serovars of *Salmonella*, while the resistance percentage of AZI and IMP were the quite low. **(B)** The composition of different serogroups. The O:4(B) serogroup consists of *S*. Derby, *S*. Typhimurium and *S*.I 4, [5],12:i:-; the O:3,10(E1) serogroup consists of serovar London, Anatum and Uganda. The O:7(C1) serogroup consists of *S*. Livingstone, *S*. Rissen, *S*. Infantis and *S*. Mbandaka. The O:B (C2–C3) serogroup consists of *S*. Goldcoast. **(C)** The distribution of the strains in different serogroups. The percentage of strains in different serogroups was as follows: O:4(B), 55%; O:7(C1), 22%; O:3,10(E1), 21%; O:B(C2–C3), 2%. **(D)** The distribution of antimicrobial resistance in four serogroups in *Salmonella*. The resistance percentage in O:3,10(E1) and O:B(C2–C3) serogroups were fairly high.

The heatmap of the antimicrobial resistance in different serovars and serogroups was shown in Figure 3. The resistance was higher than 95% toward TET, TST and CHL in all serovars of *Salmonella*. While the resistance against IMP and AZI was low in most of the serovars (Figure 3A). Anatum, Goldcoast and Uganda serovars were considered to be the highest antimicrobial resistant serovars among all serovars. As shown in Figure 3B, the 105 strains were classified into four serogroups. The O:4(B) serogroup consists of serovars from Derby, Typhimurium and I 4, [5],12:i:-; the O:3,10 (E1) serogroup consists of London, Anatum and Uganda. The O:7 (C1) serogroup consists of Livingstone, Rissen, Infantis and Mbandaka. The O:B (C2–C3) serogroup consists of Goldcoast. The distribution of the serogroups in 105 samples was as follows: O:4 (B) serogroup 55%, O:7 (C1) serogroup 22%, O:3,10 (E1) serogroup 21%, and O:8 (C2–C3) serogroup 2% (Figure 3C). The resistance percentage in O:3,10 (E1) and O:B (C2–C3) serogroups were extremely high, while the O:4(B) and O:7(C1) serogroups were susceptive to 7 of the 14 different antimicrobials (Figure 3D).

### Genotyping by Multilocus Sequence Types (MLST)

**Figure 4:**
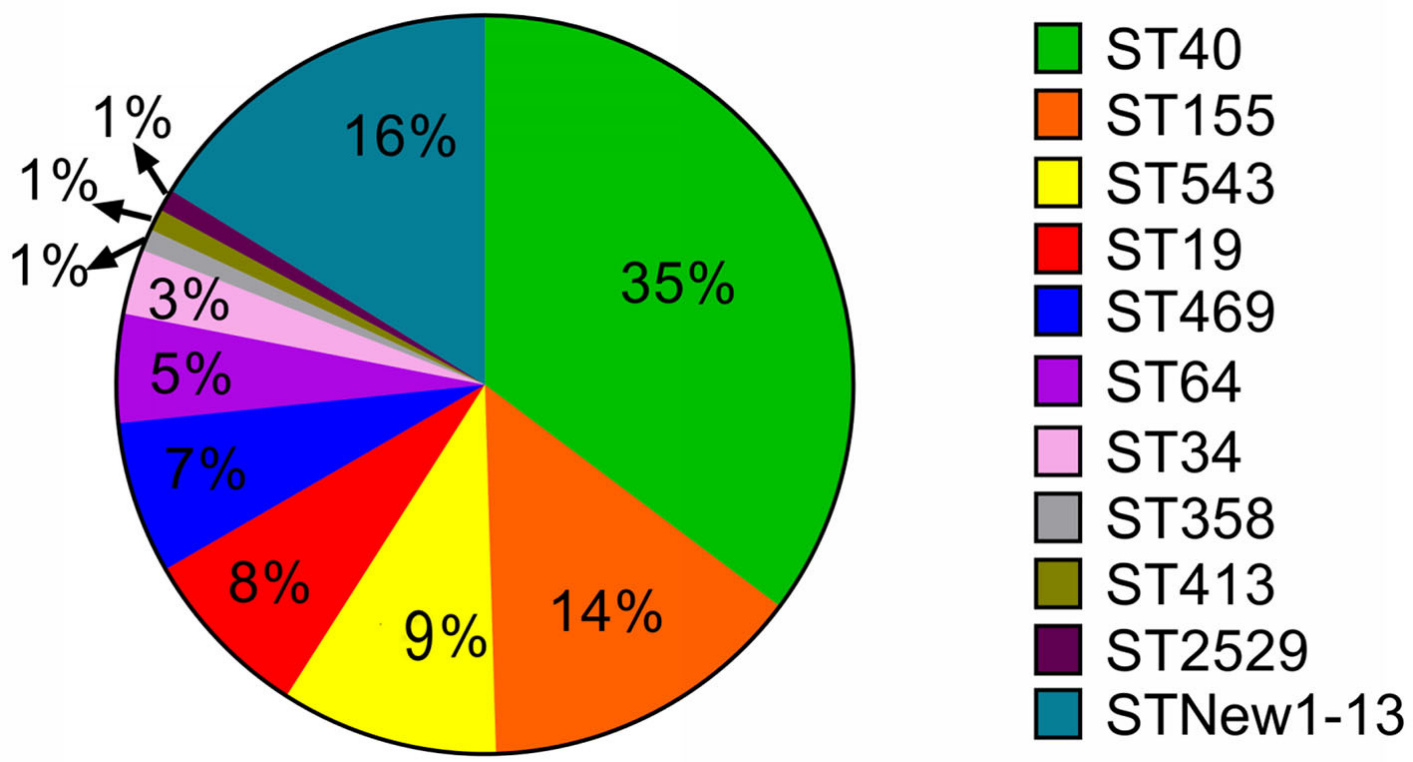
The distribution of Multilocus-sequence typing (MLST) type among 105 *Salmonella* isolates. Over half of these strains were ST40 and ST155 types. We also found 13 new sequence types in 17 strains. The new sequence types were named as MLST New 1–13 accordingly.

With further genomic analysis, we found that all these strains belonged to eleven sequence types. Half (49.5%) of the strains were found to be belonged to ST40 (35%) and ST155 (14%) (Figure 4). Our results also showed that 90% of *S*. Derby strains were belonged ST40 and only 10% were belonged to four new sequence types, including ST new 1 (three strains), ST new 10 (one strain), ST new 11 (one strain) and ST new 13 (one strain). Besides, ST155 and ST543 were the predominated STs in *S*. London and *S*. Livingstone, respectively. The serovar London had two new serovars which were named ST new 7 and ST new 8. All *S*. Rissen strains were belonged to ST469. ST19 was considered to be the dominated genotype in *S*. Typhimurium and *S*. I 4, [5]12:i:-. Similarly, the serovar Typhimurium had two new sequence types which were named ST new 5 and ST new 6. The newly found sequence type, in serovars Infantis, Livingstone, Mbandaka and Uganda were named ST new 3, ST new 2, ST new 12 and ST new 4, respectively.

### Plasmidome of *Salmonella* Isolates

**Figure 5:**
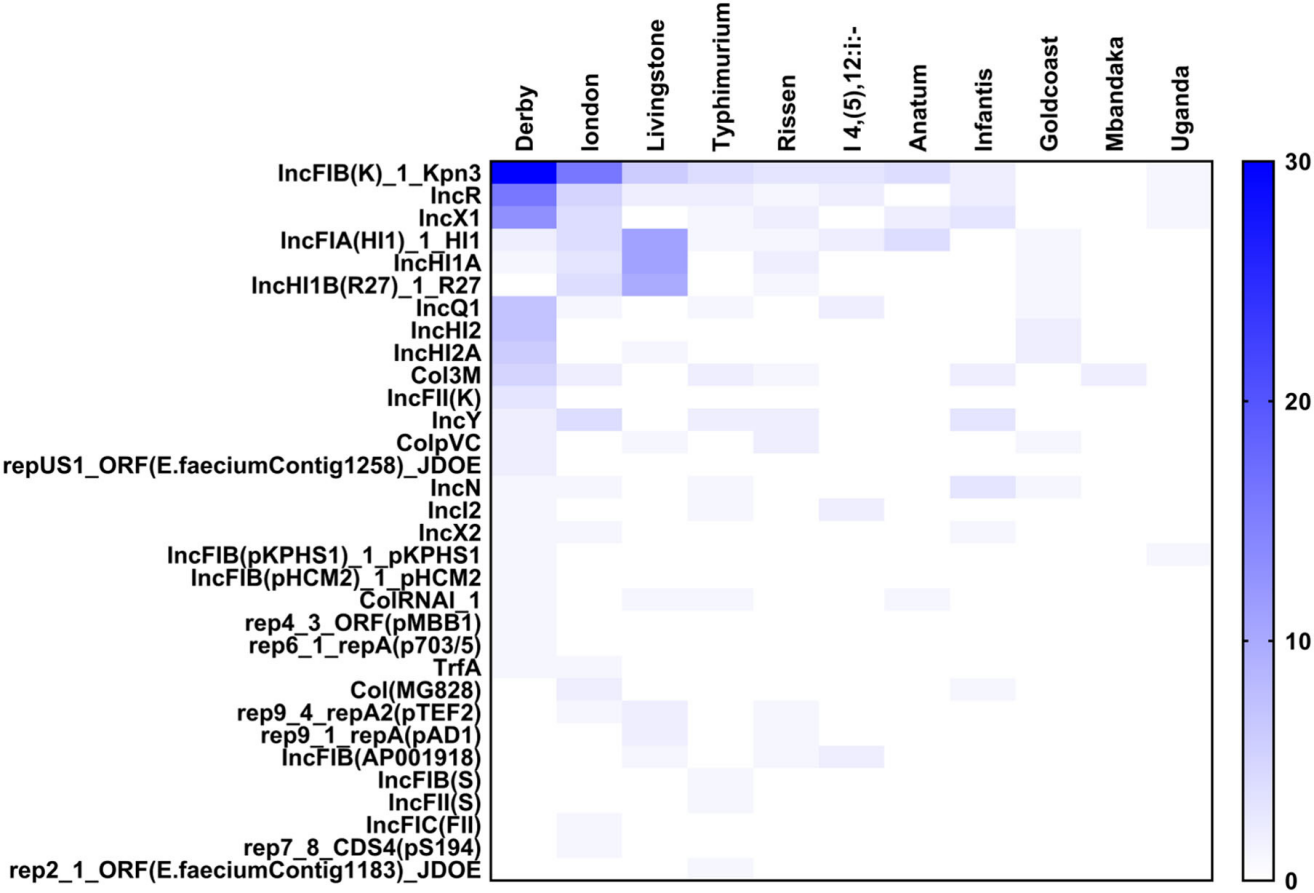
The heatmap of plasmids distribution in different *Salmonella* strains. There were 33 different plasmids in *Salmonella* strains. The *S*. Derby and *S*. London had largest number of plasmids with different kinds. The IncFIB(K)-1-Kpn3 plasmid accounted for the highest proportion in Derby and London.

**Figure 6:**
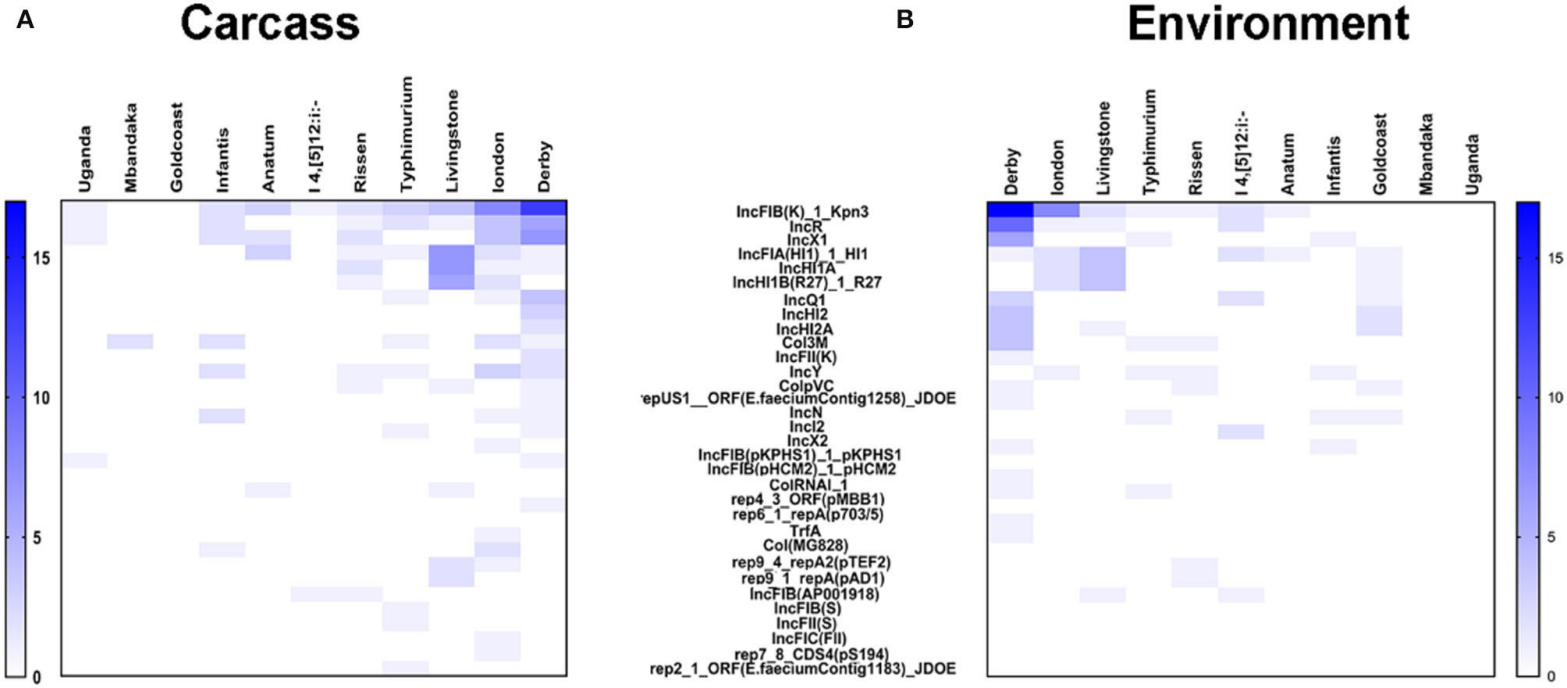
The heatmap of plasmids distribution in 105 *Salmonella* strains with different origin. **(A)** the heatmap of plasmids distribution in 105 *Salmonella* strains which were originated from animals. *S*. Derby had the largest number of plasmids with different kinds. The largest percentage of plasmids in *S*. Derby were IncFIB(K)-1-Kpn3 (76%). **(B)** the heatmap of plasmids distribution in 105 *Salmonella* strains which were originated from environment. The largest percentage of plasmids in *S*. Derby were also IncFIB(K)-1-Kpn3 (63%).

The plasmids replicons harbored in *Salmonella* isolates were analyzed using plasmid finder. As shown in Figure 5, *S*. Derby and *S*. London had a large number of different types of plasmids; however, the numbers of plasmids in *S*. Mbandaka and *S*. Uganda was low. The IncFIB(K)-1-Kpn3 plasmid accounted for the highest proportion in *S*. Derby (68%) and *S*. London (94%). Also, the IncR (36%) and IncX1 (25%) plasmids were common in *S*. Derby. Moreover, we analyzed the plasmids distribution in strains obtained from different sources. As shown in Figure 6, the *S*. Derby had the highest number of different types of plasmids, irrespective of the source (pig carcass or the environment). The IncFIB(K)-1-Kpn3 plasmid was predominant in 76 and 62% of *S*. Derby isolated from pig carcass and environment, respectively. The *S*. Goldcoast strains isolated from pig carcass had no plasmids, whereas plasmid existed in strains isolated from the environment. The *S*. Uganda and *S*. Mbandaka strains isolated from the environment had no plasmids.

### Resistome of *Salmonella* Isolates

The antimicrobial resistant genes of the strains were analyzed to explore the underlying mechanism responsible for the bacterial antimicrobial resistance. As shown in Supplementary Figure 1A, 107 different antimicrobial resistant genes were identified in 105 strains. These genes were classified into ten classes according to antimicrobial drug classes. These ten different classes were as follows: aminoglycoside, beta-lactam, polymyxin, macrolide-lincosamide-streptogramin, phenicol, sulphonamide, tetracycline, trimethoprim, fluoroquinolone and fosfomycin. Among all isolated strains, we found that *tet*(A) gene which confers resistant to tetracyclines was the highest prevalent gene with prevalence reach to (95%). Twenty-six resistant genes of beta-lactam class, in addition to eleven plasmid-mediated quinolones-resistant genes have been detected amongst the isolated strains. The widely spreading of these critical resistant genes in the strains pose a great public health concern. Our results also showed that *S*. Derby strains and O:4(B) serogroup had the highest prevalence of the antimicrobial resistant genes among the other groups and serovars with prevalence % as follows: *fos*A7 (97%), *tet*(A) (95%), *oqx*A (90%), *oqx*B (84%), *qnr*S1 (70%), *sul*2 (50%), and *dfr*aA12 (50%). With further analysis colistin resistant (*mcr*-1) gene was identified also in five strains, including two strains of *S*. Derby, two strains of I 4, [5],12:i:- and one *S*. Typhimurium strain. Of the antimicrobial resistant genes in strains isolated from different source, we found that the dominant antimicrobial resistance genes in *S*. Derby isolated from the pig carcasses were as follows: *bla*SHV-187 (41%), *sul*2(64%), *tet*(A) (100%), *dfr*aA12 (52%), *oqx*A (94%), *oqx*B (82%), *qnr*S1 (88%), and *fos*A7 (100%). The dominant antimicrobial resistance genes in O:4(B) serogroup isolated from the pig carcasses were as follows: *bla*SHV-187 (43%), *sul*2 (73%), *tet*(A) (100%), *dfr*aA12 (65%), *oqx*A (86%), *oqx*B (73%), *qnr*S1 (91%), and *fos*A7 (78%). Besides, the dominant antimicrobial resistance genes in *S*. Derby isolated from the environment were as follows: *bla*SHV-187 (40%), *sul*2 (40%), *tet*(A) (92%), *dfr*aA12 (48%), *oqxA* (88%), and *oqxB* (85%). The dominant antimicrobial resistance genes in O:4(B) serogroup isolated from the environment were as follows: *bla*SHV-187 (40%), *sul2* (48%), *tet(A)* (91%), *dfraA12* (54%), *oqxA* (77%), *oqxB* (74%), *qnrS1* (68%), and *fosA7* (80%) (Data not shown).

## Discussion

China produces and consumes about 500 million heads per annum of the global pork demand (28). Jiangsu is one of the largest pigs producing provinces of China. Pigs and pork from Jiangsu are also distributed to other adjacent provinces like Zhejiang, Shanghai, Anhui, and Shanxi or to the south as far as Guangdong. These distribution channels also aid as a channel for the spread of pathogens as well as antibiotic-resistant strains from the farms to the distributors and the consumers. Thus, the prevalence of the pathogen in Jiangsu pig/pork not only has a local but also widespread regional consequence. Frequent earlier studies have reported on the spatiotemporal variation of the prevalence of salmonellae on different types of meat, foods, and animals across China (13, 14, 32, 33). These studies suggested that pathogenic bacteria from animals could transmit AR determinants to strains isolated from humans.

The main findings of this study are as follows: (1) *S*. Derby was the dominant serovar among the 105 isolates, and most of the *S*. Derby strains were isolated from the environment. (2) All the isolated strains were multidrug resistance, regardless of the source and serovars. The isolates showed a higher percentage of resistance toward antimicrobials TET (95.4%) and TST (90.9%). (3) The most severe multidrug resistance serovar was Uganda, which was resistant to six types of antimicrobials and the most severe multidrug resistance serogroup was O:3,10 (E1). (4) IncFIB(K)-1-Kpn3 plasmid replicon was found to be the dominant plasmid type in *S*. Derby was regardless of the source. Nevertheless, only by using whole genomic sequencing approach and combined with the sophisticated bioinformatic pipeline, we are able to address the resistome and plasmidome of particular diversified bacterial population, which provide a new way for conducting the foodborne pathogens and epidemiology in China.

*Salmonella* is one of the four major global causes of diarrheal diseases. Pork products are the main source of *Salmonella* infection and slaughterhouses provide ample opportunities for *Salmonella* growth and proliferation with contamination arising from the environment, animal carcasses, utensils etc. The prevalence of *Salmonella* was quite different in slaughterhouses in different countries. In our study, we observed *S*. Derby as the dominant serovar. In Belgium and France, the dominant serovar was *S*. Typhimurium and *S*. I 4,5,12: i:-, respectively (34, 35). In China, the *Salmonella* prevalence was consistent in farms and slaughterhouses, and *S*. Derby was the dominant serovar (32), which was considered one of the top three most common *Salmonella* isolated from patients with diarrhea (36) worldwide.

Although numerous studies have investigated the prevalence and the antimicrobial resistance of *Salmonella* in the pig production chain, the underlying molecular mechanism still remains unclear. Nowadays, whole genome sequencing has become a routine and useful approach for risk assessment (37). However, this application has not yet started in China. Therefore, here we investigated the antimicrobial resistant gene and plasmid profiles by using genome sequencing approach to uncover the molecular level mechanism of antimicrobial resistance.

In our study, all the isolates were considered to be multidrug-resistant (MDR). This number was quite alarming since the MDR rate was reported 44.04 and 73.2% in studies conducted in 2017 and 2018, respectively (32). We speculated that the rapid increase in the MDR rate was due to the large and long-term antimicrobials abuse in pig breeding (38–40). Besides, all isolates were more resistant to tetracycline, chloramphenicol, ampicillin, sulfamethoxazole, and cephalosporins. These antibiotics considered the most common groups of antimicrobials used in commercial pigs farming in China (41). The presence of a higher degree of resistance to antimicrobials commonly and frequently used in farm animals certainly raises the red alarm.

We also noticed that the resistance to the most of the antimicrobial resistance rate was higher among the isolates originated from pig carcasses than the environmental originated isolates, which indicated that the main source of the isolates was the pigs and mostly the environment was cross contaminated during the current slaughter procedures.

Resistance to quinolones and beta-lactams antibiotics which was conferred due to the presence of the beta-lactam and the plasmid-mediated quinolones-resistant genes were also identified in both pig carcasses and environmental-originated isolates which poses a great public health concern, as both of them are considered critical antibiotics and also quinolones are currently preferred as the first choice of drugs for the treatment of invasive enteric salmonellosis (14). Furthermore, we detected *mcr*-1 gene in five strains, including two strains of *S*. Derby, two strains of I 4, [5],12:i:- and one *S*. Typhimurium strain which was much higher than the previous reports (42). Previous reports showed that the prevalence of *mcr*-1 gene was quite infrequent in *Salmonella*. Among the 12,053 *Salmonella* isolates in China (2006–2016), the *mcr*-1 gene was found only in 37 strains, out of which 35 strains were *S*. Typhimurium, and the remaining two strains were *S*. Enteritidis and *S*. Wandsworth (43, 44).

Even though the antimicrobial resistant genes, that responsible for the phenotypic antimicrobial resistant, were detected in the bacterial genome, the presence of these genes do not necessarily confer phenotypic resistance, and the absence of antimicrobial resistance genes does not suggest the phenotypic susceptibility (45). The phenomenon of AMR is not just related to the mere presence or absence of resistance genes. Other mechanisms such as enzyme activation, target modification/protection, regulation of AMR gene expression, or even change in the cell wall charge play some important roles in the AMR. So, when compared with just the AMR genes, some degree of discordance is expected. Because of such multiple variables and manifold association of genotypic and phenotypic data (46), a comparison of genotype-phenotype should give a better and complete picture. We also noticed that the dominant plasmid was IncFIB(K)-1-Kpn3. Most of the S. enterica isolates in this study that carried the IncFIB(K)-1-Kpn3 plasmids replicon were multidrug-resistant, which is consistent with the findings described previously (47–50). We also observed that IncFIB(K)-1-Kpn3-positive strains carry more antimicrobial resistance than isolates with other replicon types. So, we could expect that IncFIB(K)-1-Kpn3 might play a vital role in the antimicrobial resistant genes-carrying S. enterica isolates dissemination through the pig-borne products.

Additionally, our study demonstrated the direct genomic sequence could provide sufficient contamination information, i.e., type of *Salmonella*, antimicrobial resistant determinant, companion plasmids, within the pork production system. Future investigation should study the prevalence of *Salmonella* infection in humans in Jiangsu, and study the possible *Salmonella* as well as antimicrobial resistance transmission from food animals, animal-borne food, and humans in Jiangsu Province, China.

In conclusion, the present study provided a comprehensive study on the prevalence of *Salmonella* in pig slaughterhouses in Jiangsu province, China. We reported that all isolated strains showed MDR phenotype and showed more resistant to tetracycline, chloramphenicol, ampicillin, sulfamethoxazole, and cephalosporins. S. Derby serovar was found to be the most prevalent serovar among the serovars with high prevalence in the environmental samples. Additionally, *tet(A)* gene which confers resistant to tetracyclines was the highest prevalent gene. Besides, beta-lactam, and plasmid mediated- quinolones and colistin resistant (*mcr*-1) resistant genes have been also detected among the isolated strains. IncFIB(K)-1-Kpn3 was also found to be the most prevalent detected plasmid replicon among the isolates and coexisted with the large number of antimicrobial resistance genes. Therefore, there is an urgent need to avoid antimicrobial abuse and develop new antimicrobials. These findings uncovered the prevalence of *Salmonella* in slaughterhouses in Jiangsu, China, and also provided essential knowledge to guide rational antimicrobials usage if appropriate.

## Data Availability Statement

The original contributions presented in the study are included in the article/Supplementary Material, further inquiries can be directed to the corresponding author/s.

## Ethics Statement

No ethical approval was deemed necessary for this study. Oral agreement and permission were obtained from the farmers as well as the slaughterhouse manager before sampling.

## Author Contributions

WC: writing the original draft, refined and reorganized the data and its presentation. QL, WC, and ME: conducting the experiments and aiding with the writing. HP: data analysis. LW, CZ, BZ, XX, DL, XY, XH, and HL: sampling, sample preparation, and methodology. MY and YL: conceptualization, funding acquisition and project administration. All authors contributed to the article and approved the submitted version.

## Conflict of Interest

The authors declare that the research was conducted in the absence of any commercial or financial relationships that could be construed as a potential conflict of interest.

## Supplementary Material

Supplementary Figure 1 The heatmap of the resistance genes in different serovars and serogroups in *Salmonella*. **(A)** the heatmap of the resistance genes in different serovars in *Salmonella*. The 107 antimicrobial resistance genes were classified into ten different categories. The most abundance antimicrobial resistance genes were for aminoglycosides and beta-lactams. **(B)** the heatmap of the antimicrobial resistance genes in different serogroups in *Salmonella*. The dominant antimicrobial resistance genes in O:4(B) serogroups *Salmonella* were *acc*(6′)-Iaa, *bla*SHV-187, *sul, tet*(A), *dfr*A12, *oqx*A, and *fos*A.
