# Supplementary figures and images for: Characterization of Salmonella Resistome and Plasmidome in Pork Production System in Jiangsu, China
Source: Front Vet Sci. 2020 Sep 11;7:617. doi: 10.3389/fvets.2020.00617 (PMC7517575; doi:10.3389/fvets.2020.00617)

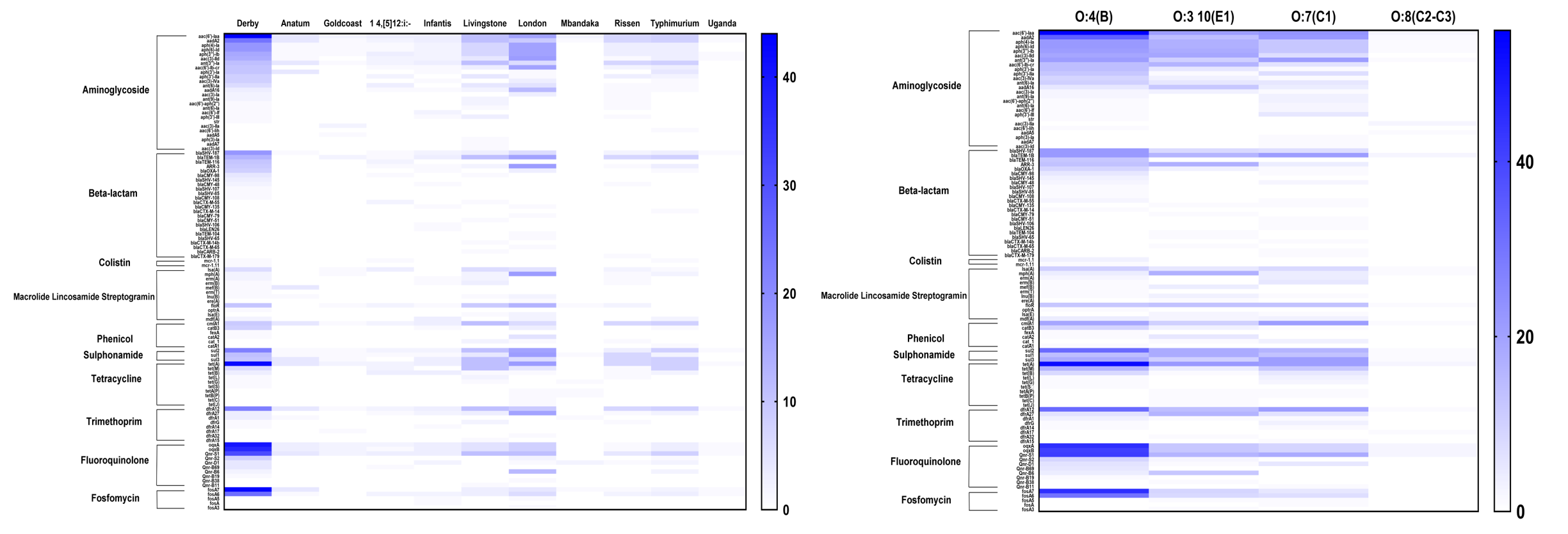

Supplement: Supplementary Figure 1 — The heatmap of the resistance genes in different serovars and serogroups in Salmonella. (A) the heatmap of the resistance genes in different serovars in Salmonella. The 107 antimicrobial resistance genes were classified into ten different categories. The most abundance antimicrobial resistance genes were for aminoglycosides and beta-lactams. (B) the heatmap of the antimicrobial resistance genes in different serogroups in Salmonella. The dominant antimicrobial resistance genes in O:4(B) serogroups Salmonella were acc(6′)-Iaa, blaSHV-187, sul, tet(A), dfrA12, oqxA, and fosA. [file Image_1.TIF]
